# Unexplained Coma and Sudden Death in Psychiatric Patients Due to Self-Induced Water Intoxication: Clinical Insights and Autopsy Findings From Two Fatal Cases

**DOI:** 10.7759/cureus.79813

**Published:** 2025-02-28

**Authors:** Stefano Pini, Accursio Raia, Barbara Carpita, Bendetta Nardi, Matteo Benvenuti, Andrea Scatena, Marco Di Paolo

**Affiliations:** 1 Department of Clinical and Experimental Medicine, University of Pisa, Pisa, ITA; 2 Department of Medicine, Surgery, Neuroscience, and Forensic Medicine, University of Siena, Siena, ITA; 3 Department of Legal Medicine, University of Pisa, Pisa, ITA

**Keywords:** autopsy findings, fatal water intoxication, psychiatric patients, sudden death, unexplained coma

## Abstract

Self-induced water intoxication is a life-threatening condition caused by excessive water intake that surpasses renal excretion capacity, resulting in hypotonic hyponatremia. This acute imbalance leads to cerebral and pulmonary edema, neurological deterioration, and potentially fatal outcomes. Psychiatric disorders such as schizophrenia and postpartum psychosis are significant contributors, often driving these behaviors through unique psychopathological mechanisms exacerbated by inadequate patient supervision. This study presents two fatal cases. The first involves a 42-year-old woman with chronic schizophrenia and psychogenic polydipsia, whose condition progressed gradually, allowing partial therapeutic intervention. The second describes a 28-year-old woman with postpartum psychosis and compulsive water drinking linked to religious delusions, whose condition deteriorated rapidly, leading to cardiopulmonary arrest shortly after admission. Both cases highlight the connection between psychiatric disorders and severe hyponatremia (<120 mmol/L), resulting in irreversible brain damage and sudden death. Autopsy findings revealed diffuse cerebral edema, pulmonary congestion, and diffuse axonal injury with reactive astrogliosis, demonstrating the severe impact of electrolyte imbalances on neuronal damage. Neuropathological findings, such as ubiquitin-positive axonal swellings and astrocytic activation, underscore the critical role of ionic homeostasis disruption in bridging clinical and autopsy observations. These cases highlight the importance of early recognition of psychogenic polydipsia and compulsive water-drinking behaviors, particularly in high-risk psychiatric patients. Preventative strategies should include routine electrolyte monitoring, caregiver education, and proactive management of psychiatric disorders. Critically, water intoxication must always be considered among the possible causes of unexplained coma or sudden death in psychiatric patients, stressing the need for clinical vigilance and accurate postmortem assessment to improve prevention.

## Introduction

Self-induced water intoxication is a potentially life-threatening condition that results from excessive water intake, surpassing the kidneys' ability to excrete it. This leads to hyponatremia, a critical condition where sodium levels in the blood drop dangerously low. Sodium is a vital electrolyte that helps regulate water balance inside and outside of cells, maintaining normal cellular function and volume. When the concentration of sodium in the bloodstream becomes too low, water moves into cells to restore balance. This shift is particularly harmful in brain cells, causing cerebral edema, or swelling of the brain, as the cells absorb too much water [1]. This swelling puts pressure on the brain and can lead to severe neurological complications such as seizures, coma, or death. Hyponatremia can be caused by a variety of factors, but one of the most common in certain psychiatric disorders is excessive water consumption. This condition is often seen in people with psychiatric disorders like schizophrenia, bipolar disorder, and neurodevelopmental disorders, including autism and intellectual disabilities [2-4]. These conditions may drive abnormal water-drinking behaviors through distinct mechanisms such as altered thirst sensation or compulsive behaviors. Additionally, the risk of water intoxication is increased in individuals who are isolated or inadequately supervised, as they may not be monitored for abnormal drinking patterns.

The pathophysiology of water intoxication and its severe neurological effects stems from the rapid shift of water into brain cells as a result of low sodium levels. The brain cells swell, causing increased intracranial pressure, which can damage brain tissue. Over time, this swelling may lead to reactive astrogliosis - a process where astrocytes, the cells that support neurons, react to injury by multiplying and producing proteins to protect the brain. However, this reaction can further impair brain function. Another potential consequence of prolonged cerebral edema is the formation of ubiquitin-positive axonal swellings. These are signs of neurodegeneration and indicate the accumulation of damaged proteins within neurons, which can lead to long-term brain damage.

Clinically, self-induced water intoxication typically presents as rapidly progressing hyponatremic encephalopathy. Early symptoms may include blurred vision, fatigue, tremors, and muscle cramps, as well as changes in mental status such as confusion. If the condition worsens, seizures, coma, and significant brain swelling may occur, often resulting in fatal outcomes. The rapid progression of symptoms underscores the critical need for early recognition and intervention to prevent death.

The neuropathology of water intoxication remains poorly understood. Although conditions like central pontine myelinolysis and brainstem herniation have been documented, these are sometimes attributed to improper management, such as overly rapid correction of hyponatremia. In many cases, no definitive brain lesions are found to fully explain death or long-term neurological damage. Autopsy findings in cases of self-induced water intoxication are often non-specific but typically show signs of cerebral edema and pulmonary edema (fluid accumulation in the lungs). These findings reflect the damage caused by the low sodium levels but do not provide definitive evidence for the cause of death [5-8]. While cerebral edema and pulmonary edema are commonly reported, these findings alone cannot definitively establish water intoxication as the cause of death. Indeed, cerebral edema, while being one of the most consistent findings in cases of water intoxication can also be seen in a variety of other conditions such as trauma, anoxia, infections, and metabolic disorders. Similarly, pulmonary edema can also occur in patients with cardiac failure, acute respiratory distress syndrome (ARDS), or severe infections such as pneumonia. Moreover, sepsis and severe infections, especially those with an encephalitic component, can cause both cerebral and pulmonary edema, alongside altered mental status. Additionally, the neuropathology associated with water intoxication, such as central pontine myelinolysis, although documented, is often attributed to inappropriate management, like overly rapid correction of hyponatremia, rather than the intoxication itself. Central pontine myelinolysis, a condition involving the breakdown of myelin in the brainstem, has also been observed in some cases. However, it is often linked to improper management, such as correcting sodium levels too quickly. In most cases of water intoxication, no specific brain lesions are identified, making the diagnosis difficult. In conclusion, autopsy findings of water intoxication can overlap with those of other conditions such as central pontine myelinolysis, dehydration, drug toxicity, infections, cardiac pathology, and metabolic disorders, thus it is important to consider a range of differential diagnoses when interpreting them.

The lack of sufficient clinical data and the under-recognition of this phenomenon suggest that self-induced water intoxication is often underdiagnosed, particularly in cases of sudden death among psychiatric patients who live alone or receive inadequate monitoring. In this report, we describe two fatal cases of self-induced water intoxication: one in a patient with schizophrenia, and another in a woman with postpartum psychotic depression. By analyzing the psychopathological presentations and autopsy findings, we aim to provide insights into this condition. We emphasize the importance of strongly suspecting water intoxication in cases of sudden death or unexplained coma in individuals with severe psychiatric disorders.

## Case presentation

Case 1

**Figure 1:**
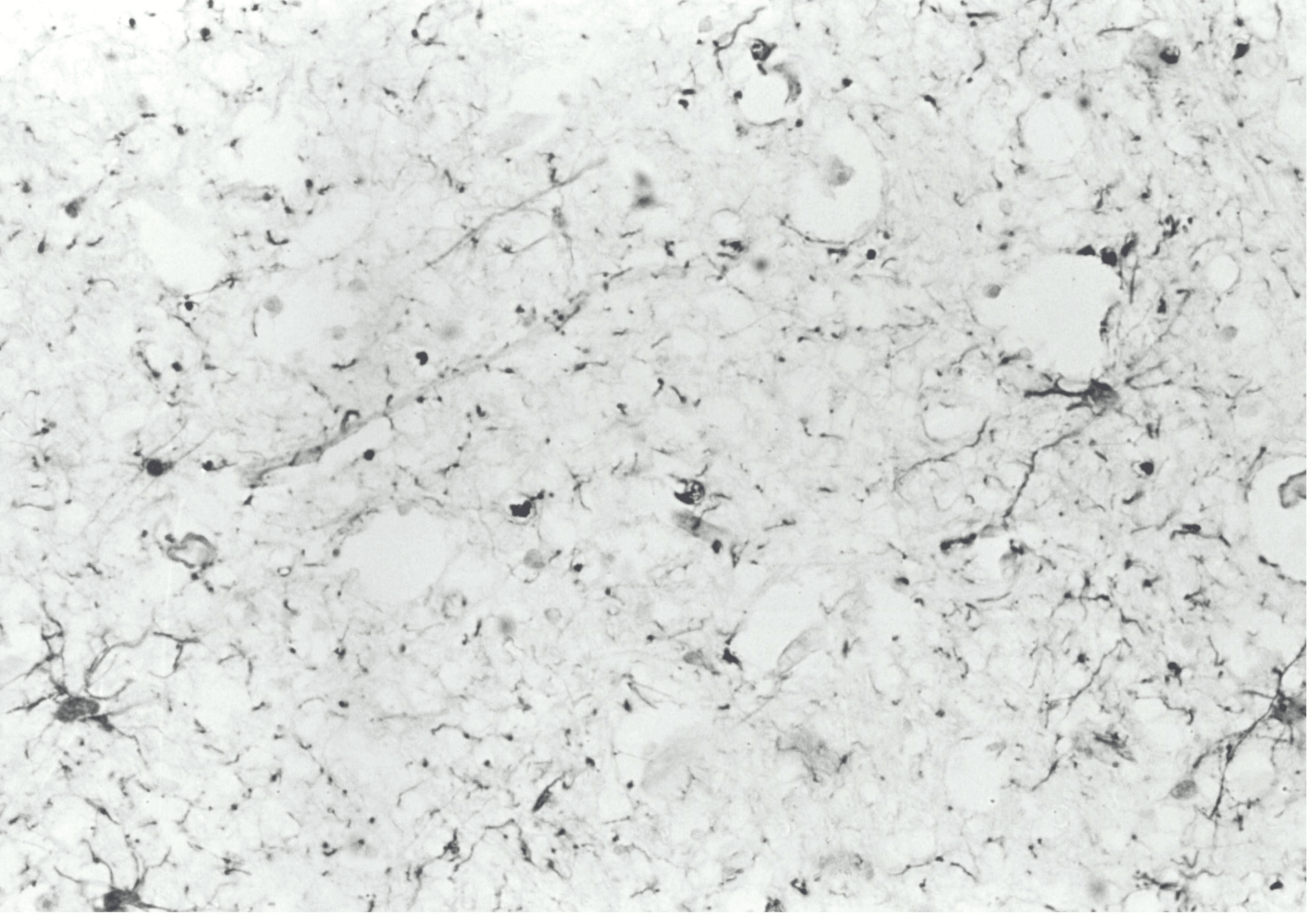
Case 1: Cerebral cortex (x450) showing GFAP-positive cells at higher magnification. GFAP: glial fibrillary acidic protein.

**Figure 2:**
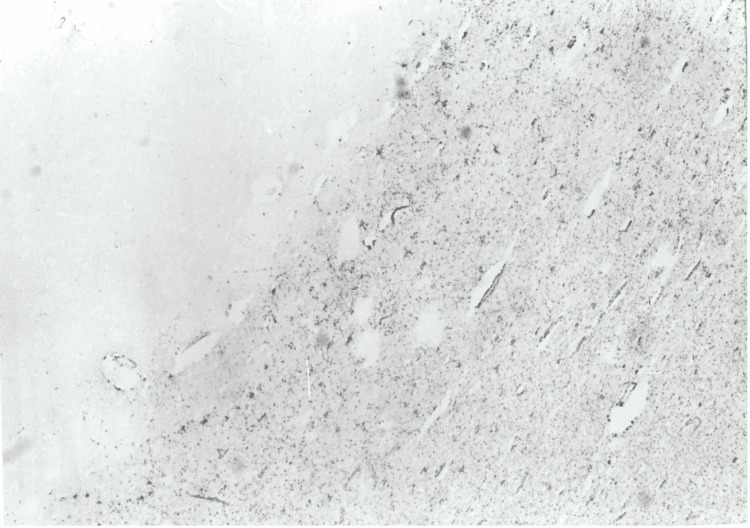
Case 1: Frontal section (GFAP staining, x150) showing astrocytosis of the white matter, more accentuated in the subcortical region. GFAP: glial fibrillary acidic protein.

A 42-year-old woman diagnosed with schizophrenia at age 18 and with a history of alcohol use disorder was found comatose by her mother, who found several empty bottles of mineral water in her room. The patient had a family predisposition to schizophrenia, as the mother was also affected. In the past, occasional consumption of large amounts of water secondary to delusional ideas of intestinal contamination had been observed, but without obvious clinical consequences. The patient was on chronic treatment with antipsychotics, but no specific details of adherence to therapy in the weeks prior to admission were documented. No recent reports of alcohol abuse were present. Upon arrival at the hospital, the patient was in a deep coma with bilateral mydriasis and no corneal and photometric reflexes. The vital signs at admission were as follows: arterial pressure of 100/70 mmHg, heart rate of 82 bpm, respiratory rate of 18 acts/min, and oxygen saturation at 95% in ambient air. No signs of external trauma were noted. Laboratory tests revealed severe hyponatremia (117.5 mEq/L) and hypokalemia (3.0 mEq/L), with creatinine at 0.7 mg/dL, and plasma osmolality at 251 mOsm/kg, confirming a picture of water intoxication. Liver tests were normal. Mannitol (0.5 g/kg) was given to reduce cerebral edema, with a subsequent abundant diuresis, and the patient was also treated with glycerol (10% oral solution) for intracranial hypertension management. Direct treatment of hyponatremia with hypertonic saline solution was not attempted, considering the risk of pontine myelinolysis syndrome. An electrocardiogram (ECG) revealed a prolonged QT interval (QTc: 520 ms), probably related to both hypokalemia and chronic treatment with neuroleptics. A computed tomography (CT) of the brain showed diffuse cerebral edema without signs of herniation. Specifically, the CT report showed "complete and widespread flattening of the brain sulci associated with obliteration of the cisternal spaces, particularly at the level of the pons-midbrain. Apparent compressive signs, especially affecting the temporal and occipital horns and at the level of the third ventricle, which is barely visible. A relative increase in density is noted in the area of the tentorium and the insertion of the great falx in the posterior aspect. Diffuse encephalic suffering due to edema." Despite the treatments, an electroencephalogram (EEG) taken 24 hours later showed no brain activity, indicating irreversible neurological damage. The patient died 36 hours after admission. The toxicology tests performed on blood and urine were negative for alcohol, amphetamines, barbiturates, cannabinoids, benzodiazepines, cocaine, methadone, and opiates. The absence of drugs or toxic substances excludes other causes of coma, reinforcing the diagnosis of water poisoning. Figures 1, 2 show the immunohistochemical images obtained during the autopsy.

Case 2

**Figure 3:**
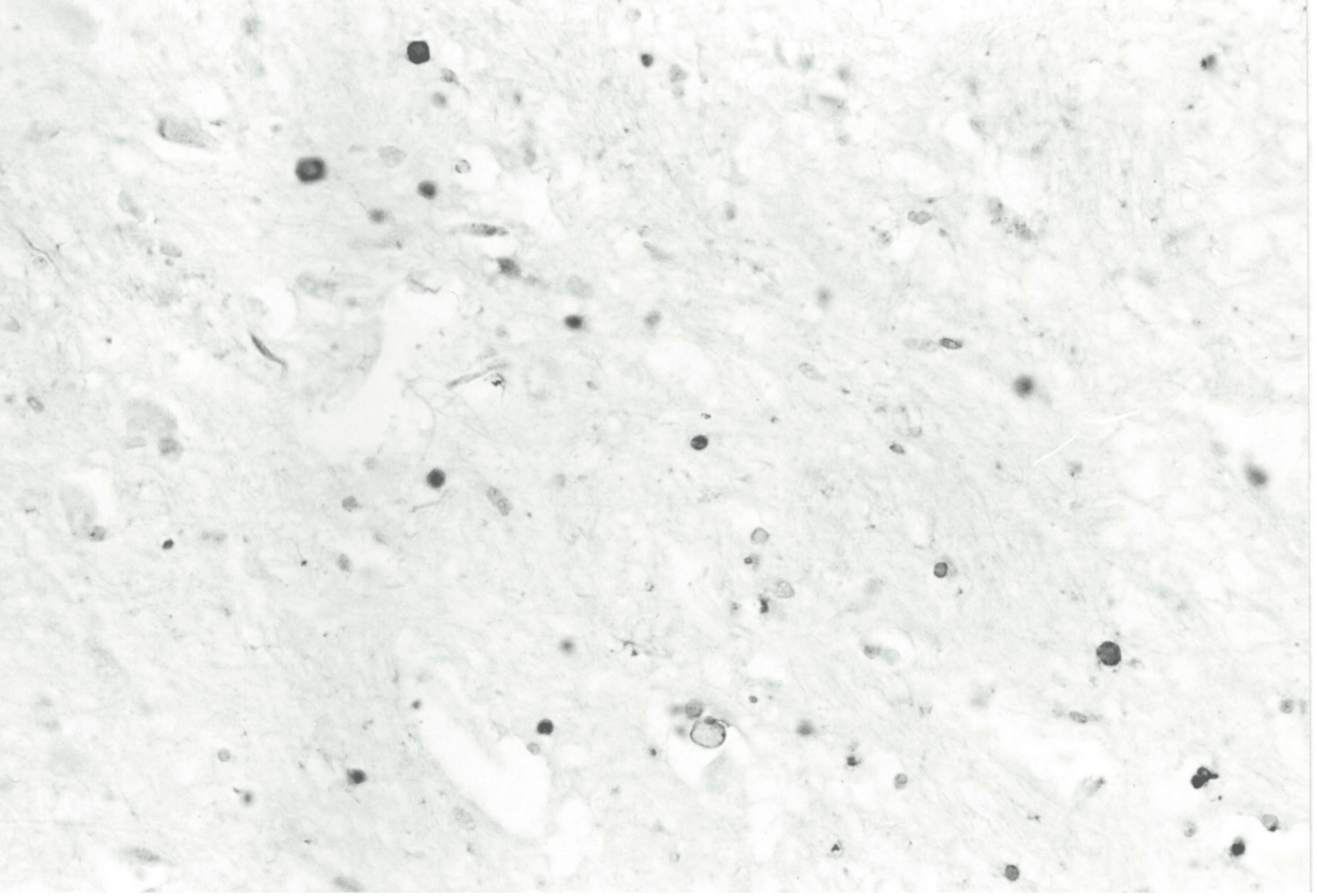
Case 2: White matter (ubiquitin staining, x300) showing several positive axonal spheroids.

A 28-year-old woman, mother of a newborn, developed postpartum depression about two months after giving birth. She was initially treated with lithium therapy but stopped taking medication against medical advice about three weeks before admission. The suspension was followed by the appearance of psychotic symptoms, including visual and auditory hallucinations, accompanied by religious-mystical delusions. According to her husband, the patient showed increasingly compulsive behavior, with episodes of excessive consumption of water, justified by the belief of "cleansing" from a supposed possession. At the time of admission, there were no previous documented episodes of psychogenic polydipsia. The patient was found by her husband in a state of acute delirium and psychomotor agitation. She was completely wet and held a bottle of water, continuing to drink compulsively. The husband reported that he had consumed large amounts of water in the last few days, but no obvious symptoms had appeared until then. The woman was taken to the hospital, where she was drugged with a combination of benzodiazepines and haloperidol. At the time of admission, the vital parameters of the patient were as follows: arterial pressure of 160/90 mmHg, heart rate of 90 bpm, respiratory rate of 20 acts/min, and oxygen saturation at 97% in ambient air. Physical examination showed no obvious trauma or significant abnormalities in the chest or abdomen. The patient was agitated, confused, and not cooperative. Initial laboratory tests showed severe hyponatremia (sodium = 115 mmol/L), hypokalemia (potassium = 3.2 mmol/L), and plasma osmolality of 250 mOsm/kg, consistent with an acute water intoxication picture. About an hour after admission, the patient developed sudden dyspnea, accompanied by foam coming out of her mouth and nose. Despite the rapid intervention with cardiopulmonary resuscitation, the woman died a few minutes later from cardiac arrest. During the initial treatment, no attempt was made to immediately restore hyponatremia with hypertonic saline solutions, given the rapid clinical progression. Figure 3 shows the immunohistochemical images obtained during autopsy.

Autopsy findings

The neuropathological analysis involved the brain, stored in 10% formalin for 30 days. Cross-sections of the grey and white matter, including samples of the cerebellum and brainstem, were prepared using standard paraffin inclusion techniques and analyzed with histological staining such as hematoxylin and eosin, Woelcke for myelin, Nissl, and silver impregnation (Bodian). Immunohistochemistry was conducted using the avidin-biotin method with antibodies directed against ubiquitin, glial fibrillary acidic protein (GFAP), subunits of 68, 160, and 200 kDa, and β-amyloid neurofilaments. Macroscopically, the cerebral gyrus appeared flattened, consistent with diffuse cerebral edema. Coronal brain sections showed no significant structural abnormalities. Microscopically, the motor neurons of the brain stem, in particular in the hypoglossal nucleus, were small and without recognizable Nissl bodies, while in the lower olives, there was an accumulation of lipofuscin, a sign of degenerative processes. No evidence of pontine myelinolysis was found. In the white matter of the hemispheres and brain stem, and to a lesser extent in the cortex, there were evident axonal bulges (axonal spheres), observable with Bodian staining and strongly immunoreactive for ubiquitin and neurofilaments, suggesting an interruption of axonal transport (see Figure 3). The white substance had mild microspongiosis and GFAP-positive reactive astrocytosis, particularly in the subcortical regions (see Figures 2, 3). The immunostaining for β-amyloid was negative. The macroscopic examination of the other organs showed widespread congestion and edema. The brain was severely edematous, while a massive edema was observed in the lungs. In the pericardial cavity, there was 70 ml of light yellow fluid, and the intracardiac blood appeared dark red and diluted. The other organs showed no significant gross abnormalities. Histopathology confirmed widespread congestion and edema.

In conclusion, the gross examination and the microscopic investigations performed according to protocol did not provide any valid alternative diagnoses to the diagnosis of cerebral edema.

## Discussion

Clinical discussion

These two cases illustrate clinical manifestations of acute water intoxication, a potentially fatal condition often associated with psychiatric disorders, highlighting similarities and significant differences in clinical and psychiatric contexts. In the first case, a woman with chronic schizophrenia, treated with antipsychotics, developed a water intoxication picture linked to episodes of pre-existing psychogenic polydipsia secondary to delusional ideas, with a relatively slow progression that allowed partial therapeutic interventions. In the second case, a woman with postpartum psychosis manifested compulsive water consumption behavior associated with mystical-religious delusions, which led to a rapidly progressive clinical picture culminating in cardiopulmonary arrest. Both cases highlight the relationship between psychogenic polydipsia and psychiatric disorders. Psychogenic polydipsia is well documented in patients with schizophrenia, with an estimated prevalence of 6% to 20% [9]. It is often aggravated by chronic use of antipsychotics, which can affect the regulation of thirst by altering the dopamine and serotonin circuits, particularly those that have a strong affinity for dopamine D2 receptors. Conversely, certain antipsychotics like clozapine have been observed to mitigate polydipsic behaviors in some patients [10]. In the second case, compulsive behavior was linked to postpartum psychosis, a condition associated with a high risk of delusions and self-injurious behaviors. The suspension of lithium therapy may have contributed to the onset of psychosis, amplifying the risk of dangerous behaviors such as compulsive water consumption. The pathophysiology of acute water poisoning is similar in both cases: massive intake of water led to severe hyponatremia (sodium <120 mmol/L) and cerebral and pulmonary edema. Acute hyponatremia causes osmotic movement of fluid within cells, with cellular swelling and increased intracranial pressure. This is responsible for the severe neurological symptoms observed in the first case, including coma and bilateral mydriasis, and acute respiratory manifestations in the second case, characterized by sudden dyspnea and oral and nasal foam. In the first case, mannitol and glycerol treatment aimed to reduce cerebral edema, but no direct intervention on hyponatremia with hypertonic saline solutions was attempted for fear of complications such as central pontine myelinolysis syndrome. In the second case, the rapid clinical progression did not allow a targeted intervention, contributing to the death in a very short time. A direct comparison between the two cases shows that the chronic course in the first case allowed partial management of complications, while the fulminant nature of the second made effective therapeutic interventions impossible. In the first case, compulsive behavior was already known but underestimated, while in the second, it developed acutely in association with psychotic delusions. This underlines the importance of early recognition of risk behaviors in psychiatric patients by implementing targeted preventive strategies. Understanding the pathophysiology and clinical manifestations of water poisoning is essential to prevent fatal complications and improve clinical outcomes in vulnerable patients. Finally, better integration between psychiatry, internal medicine, and emergency could facilitate early diagnosis and the timely implementation of targeted therapeutic interventions.

Discussion of autopsy findings

Neuropathological findings show diffuse axonal lesions (DAI), characterized by strongly positive axonal swellings for ubiquitin and neurofilaments, which indicate an interruption of intracellular transport [11]. These lesions are distinctive markers of significant axonal damage, often accompanied by functional alterations such as impairment of synaptic transmission [12]. The strong ubiquitin positivity suggests an early activation of non-lysosomal proteolytic systems in response to damage [13]. In addition, the GFAP-positive reactive astrocytosis observed in the white substance highlights the involvement of astrocytes in repair and compensatory processes [14].

The presence of these neuropathological findings, however, raises significant questions about the potential link between hyponatremia and axonal damage. The absence of macroscopic cortical alterations coupled with degenerative changes observed primarily in the brainstem suggests that the axonal lesions could be attributed to a global disruption of ionic homeostasis. Specifically, acute hyponatremia is often associated with a low plasma osmolality, which can lead to a dilutional shift of water into cells, resulting in cerebral edema and disruption of the blood-brain barrier. This fluid imbalance can affect the ionic gradient essential for maintaining normal cellular function, contributing to the observed axonal damage. In particular, the breakdown of ionic gradients impairs the transport of essential molecules along axons, leading to their degeneration and the dysfunction of synaptic transmission. While the exact underlying mechanism remains incompletely understood, these alterations in cellular function are consistent with other conditions that result in axonal injury, including mechanical trauma, ischemia, and global cerebral hypoxia. In fact, hyponatremia-related neuronal damage has been compared to the effects of traumatic brain injury, in which shear forces cause axonal stretch and damage. This comparison suggests that hyponatremia could potentially induce similar disruptions in the axonal cytoskeleton and intracellular transport systems [15].

In addition to water intoxication, there are various other natural causes of hyponatremia that can similarly affect the brain, resulting in similar neuropathological changes. These include kidney dysfunction that results in dilutional hyponatremia, which can exacerbate neuronal swelling and lead to brain edema, ultimately causing neuroinflammation and axonal injury; syndrome of inappropriate antidiuretic hormone secretion (SIADH) that leads to water retention and a dilution of sodium in the blood, and ultimately osmotic imbalances in the brain; and hyponatremia due to malnutrition or alcoholism, both conditions can impair the body's ability to manage water and sodium, leading to similar pathophysiological changes in the brain as seen in other causes of hyponatremia.

## Conclusions

Self-induced water intoxication represents a critical but underdiagnosed cause of sudden death and unexplained coma, particularly in psychiatric patients with underlying conditions such as schizophrenia and postpartum psychosis. Water intoxication occurs when excessive water intake overwhelms the kidneys' ability to excrete it, leading to a significant dilution of sodium levels in the bloodstream. This condition is most often associated with a hyponatremic state. The imbalance causes a disruption in cellular osmotic gradients, leading to cerebral edema, or swelling of the brain. The symptoms of water intoxication can range from mild to severe, depending on the degree of hyponatremia. Early symptoms include headache, nausea and vomiting, fatigue, confusion or disorientation, and muscle weakness or cramps. As sodium levels drop, the clinical picture may worsen with severe confusion, irritability, dizziness, restlessness, and ataxia. Ultimately, when reaching severe hyponatremia, seizures, coma, respiratory arrest, and even death can occur. Potential causes of water intoxication comprise impaired renal function, congestive heart failure, Addison’s disease, hypothyroidism, and the use of certain drugs such as thiazide diuretics and chemotherapy agents. Moreover, as previously stated, several medical conditions may present symptoms similar to those seen in water intoxication, increasing the difficulty of differential diagnosis. In this framework, to distinguish water intoxication from similar conditions, it is necessary to thoroughly investigate the patient history, focusing on fluid intake and potential underlying conditions. In cases of water intoxication, a clear history of excessive water intake is usually present. This is less likely in conditions like SIADH, where fluid retention occurs without excessive intake. Moreover, a very low sodium level strongly suggests water intoxication or severe dilutional hyponatremia, and urine sodium is also typically low because the kidneys attempt to conserve sodium in response to excessive water intake. Lastly, imaging techniques such as CT or MRI of the brain can help rule out structural causes of brain swelling (e.g., trauma, tumors, and infections) and show diffuse cerebral edema without focal lesions or abnormalities.

These cases highlight the interplay between compulsive water-drinking behaviors and acute hyponatremia, leading to severe cerebral and pulmonary edema. The associated neuropathological findings, including DAI and reactive astrogliosis, underline the significant role of electrolyte imbalances in disrupting neuronal function and brain homeostasis. It is essential to consider this diagnostic hypothesis, as cerebral edema represents a nonspecific finding, while the assessment of diffuse axonal injury necessitates immunohistochemical analyses that are not routinely utilized in standard autopsy procedures. Early recognition of polydipsia and its potential complications is essential in high-risk patients. Multidisciplinary approaches involving psychiatry, emergency medicine, and neurology are vital to improve outcomes. Preventive measures should include caregiver education, regular monitoring of serum electrolytes, and targeted interventions for psychiatric conditions. Autopsy findings, particularly those indicating cerebral edema and axonal damage, should prompt clinicians and pathologists to consider water intoxication in cases of sudden death or unexplained neurological deterioration, ensuring timely diagnosis and the implementation of preventive strategies.
